# DeepBreath—automated detection of respiratory pathology from lung auscultation in 572 pediatric outpatients across 5 countries

**DOI:** 10.1038/s41746-023-00838-3

**Published:** 2023-06-02

**Authors:** Julien Heitmann, Alban Glangetas, Jonathan Doenz, Juliane Dervaux, Deeksha M. Shama, Daniel Hinjos Garcia, Mohamed Rida Benissa, Aymeric Cantais, Alexandre Perez, Daniel Müller, Tatjana Chavdarova, Isabelle Ruchonnet-Metrailler, Johan N. Siebert, Laurence Lacroix, Martin Jaggi, Alain Gervaix, Mary-Anne Hartley, with the Pneumoscope Study Group, Florence Hugon, Derrick Fassbind, Makura Barro, Georges Bediang, N. E. L. Hafidi, M. Bouskraoui, Idrissa Ba

**Affiliations:** 1 Intelligent Global Health Research Group, Machine Learning and Optimization Laboratory, Swiss Federal Institute of Technology (EPFL), Lausanne, Switzerland; 2 Division of Pediatric Emergency Medicine, Department of Women, Child and Adolescent, Geneva University Hospitals (HUG), University of Geneva, Switzerland, Geneva, Switzerland; 3 Pediatric Emergency Department, Hospital University of Saint Etienne, Saint Etienne, France; 4 Center for Intelligent Systems (CIS), Swiss Federal Institute of Technology (EPFL), Lausanne, Switzerland; 5 Division of Pediatric Emergency Medicine, Department of Pediatrics, Inselspital, Bern University Hospital, University of Bern, Bern, Switzerland; 6 Geneva University Hospitals, Geneva, Switzerland; 7 Department of Pediatrics, Hospital da Crianca Santo Antonio, Porto Allegre, Brasil; 8 Department of Pediatrics, University Hospital Souro Sano, Bobo Dioulasso, Burkina Faso; 9 Faculty of Medicine and Biomedical Sciences, University of Yaounde 1, Yaounde, Cameroon; 10 University Children Hospital, Rabat, Morocco; 11 Faculty of Medicine, University Cadi Ayyad, Marrakech, Morocco; 12 Faculty of Medicine, University Cheick Anta Diop, Dakar, Senegal

**Keywords:** Respiratory signs and symptoms, Respiratory tract diseases, Paediatrics

## Abstract

The interpretation of lung auscultation is highly subjective and relies on non-specific nomenclature. Computer-aided analysis has the potential to better standardize and automate evaluation. We used 35.9 hours of auscultation audio from 572 pediatric outpatients to develop *DeepBreath* : a deep learning model identifying the audible signatures of acute respiratory illness in children. It comprises a convolutional neural network followed by a logistic regression classifier, aggregating estimates on recordings from eight thoracic sites into a single prediction at the patient-level. Patients were either healthy controls (29%) or had one of three acute respiratory illnesses (71%) including pneumonia, wheezing disorders (bronchitis/asthma), and bronchiolitis). To ensure objective estimates on model generalisability, *DeepBreath* is trained on patients from two countries (Switzerland, Brazil), and results are reported on an internal 5-fold cross-validation as well as externally validated (extval) on three other countries (Senegal, Cameroon, Morocco). *DeepBreath* differentiated healthy and pathological breathing with an Area Under the Receiver-Operator Characteristic (AUROC) of 0.93 (standard deviation [SD] ± 0.01 on internal validation). Similarly promising results were obtained for pneumonia (AUROC 0.75 ± 0.10), wheezing disorders (AUROC 0.91 ± 0.03), and bronchiolitis (AUROC 0.94 ± 0.02). Extval AUROCs were 0.89, 0.74, 0.74 and 0.87 respectively. All either matched or were significant improvements on a clinical baseline model using age and respiratory rate. Temporal attention showed clear alignment between model prediction and independently annotated respiratory cycles, providing evidence that *DeepBreath* extracts physiologically meaningful representations. *DeepBreath* provides a framework for interpretable deep learning to identify the objective audio signatures of respiratory pathology.

## Introduction

Respiratory diseases are a diverse range of pathologies affecting the upper and lower airways (pharynx, trachea, bronchi, bronchioles), lung parenchyma (alveoli) and its covering (pleura). The restriction of air flow in the variously sized passageways creates distinct patterns of sound that are detectable with stethoscopes as abnormal, “adventitious” sounds such as wheezing, rhonchi and crackles that indicate airflow resistance or the audible movement of pathological secretions. While there are some etiological associations with these sounds, the causal nuances are difficult to interpret by humans, due to the diversity of differential diagnoses and the non-specific, unstandardized nomenclature used to describe auscultation^1^.

Indeed, despite two centuries of experience with conventional stethoscopes, during which time it has inarguably become one of the most ubiquitously used clinical tools, several studies have shown that the clinical interpretation of lung sounds is highly subjective and varies widely depending on the level of experience and specialty of the caregiver^1,2^. Deep learning has the potential to discriminate audio patterns more objectively, and recent advances in audio signal processing have shown its potential to out-perform human perception. Many of these new advances were ported from the field of computer vision. In particular, convolutional neural networks (CNNs) adapted for audio signals have achieved state-of-the-art performance in speech recognition^3^, sound event detection^4^, and audio classification^5^. Audio is often transformed into a 2D image format during standard processing. The most common choice being a spectrogram, which is a visual representation of the audio frequency over time.

Several studies have sought to automate the interpretation of digital lung auscultations (DLA)^6–8^, with several more recent ones using deep learning models such as CNNs^9,10^. However, most studies aim to automate the detection of the adventitious sounds that were annotated by humans^11–13^. Thus, integrating the limitations of human perception into the prediction. Further, as these pathological sounds do not have specific diagnostic/prognostic associations, the clinical relevance of these approaches is limited^14^.

Instead of trying to reproduce a flawed human interpretation of adventitious sounds, directly predicting the diagnosis of a patient from DLA audio would likely learn more objective patterns, and also produce outputs that would be able to guide clinical decision making. Our study takes this approach of diagnostic prediction, and goes a step further to consider the patterns at a patient-level. This means aggregating recordings acquired at several anatomic locations on a single patient. Patient-level predictions have the potential to identify more complex diagnostic signatures of respiratory disease, such as etiology-specific patterns in anatomic and temporal distribution (for example expiratory wheezing or lobular vs diffuse pneumonia).

Most prior studies building deep learning models for diagnostic classification from DLA audio are difficult to assess or compare due to important inconsistencies in patient inclusion criteria. Many of these studies use the International Conference on Biomedical Health Informatics (ICBHI) public data set^15^, which exhibits multiple fundamental acquisition flaws that create systematic biases between the predicted labels. For instance, different diagnoses are collected in different locations, systematically different age groups or even using different stethoscopes (the identified biases in this data set are listed in Supplementary Fig. 1 and Supplementary Table 1).

Such systematic biases and limitations can allow the model to discriminate classes based on differences in background noise, which are likely much easier patterns to exploit and would thus result in seemingly excellent predictive performances. Indeed, studies on this data set report extremely high performances of 99% sensitivity when discriminating healthy from pathological breathing^16^ and 92% for the detection of Chronic Obstructive Pulmonary Disease (COPD)^17^. Other studies not using the ICBHI dataset tend to be limited to specific diseases, or single geographic locations, which risks poor generalizabilty.

These examples highlight the crucial importance of external validation and model interpretability to assess predictive robustness and rule out the possibility of confounding factors being exploited for classification. To our knowledge, no prior studies proposing diagnostic deep learning models on DLA recordings have been externally validated on independently collected data, nor has there been an effort to verify that their predictions align to physiological signals. The concept of dataset shift is becoming increasingly recognized as a practical concern with dramatic effects in performance^18^. Such as in an analogous example of diagnostic models for automated X-ray interpretation, where the performance on external test data proved to be significantly lower than that originally reported^19^.

In this study, we have made a specific effort to generate a pathologically diverse data set of auscultation recordings, that are acquired from a systematically recruited patient cohort, with standardized inclusion criteria and diagnostic protocols as well as broad geographic representation. We take several steps to detect and correct for bias and over fitting, reporting performance on independently collected external test data, computing confidence intervals and showing how the model’s predictions align with the cyclical nature of breath sounds to better interpret the clinical validity of outputs.

Lung auscultation is a ubiquitous clinical exam in the diagnosis of lung disease and its interpretation can influence care. Diagnostic uncertainty can thus contribute to why respiratory diseases are among the most widely misdiagnosed^20,21^. Improvements in predictive performance of this exam would not only improve patient care, but could have a major impact on antibiotic stewardship.

## Results

### *DeepBreath*—binary predictions

**Fig. 1:**
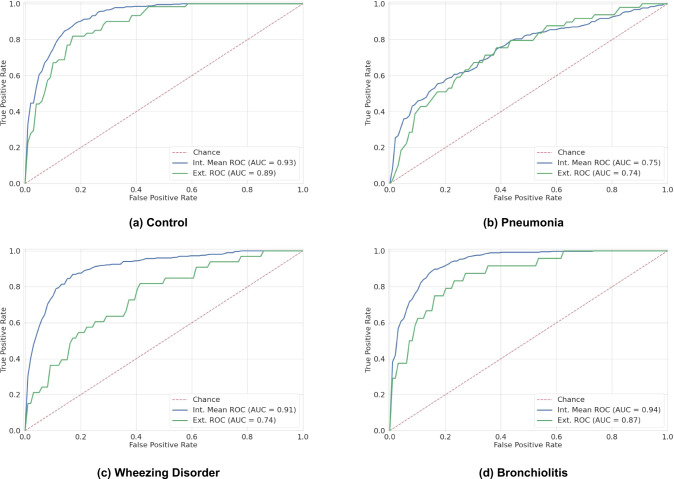
*DeepBreath* ROC curve for binary classifiers on internal and external validation data. Each panel shows the ROC curves of one binary classifier: **a** Control, **b** Pneumonia, **c** Wheezing Disorder, **d** Bronchiolitis. Every iteration of nested CV yields a different model, which produces a receiver-operating characteristic (ROC) curve for the internal test fold. The mean was computed over the obtained ROC curves. For the external validation data, predictions are averaged across the nested CV models, and a single ROC curve is computed. Internal validation is performed on various test folds from the Geneva and Porto Alegre data (blue). External validation (green) is performed on independently collected unseen data from Dakar, Marrakesh, Rabat and Yaoundé.

The four binary submodels of *DeepBreath* and the Baseline models were evaluated on both internal test folds and external validation data. The results are reported in Table 1 and 2, and Fig. 1. The *DeepBreath* model that discriminates healthy from pathological patients achieved an internal test AUROC of 0.931. This is 5% better than the corresponding Baseline model. On the external validation, it performed similarly well with an AUROC of 0.887, which is 18% better than the Baseline model. Note that the performance on the internal validation is not directly comparable to the external because the evaluation is made on individual models trained on a subset of the internal set in the former, and on an ensemble of these models in the latter. Significant differences in performance were seen among disease classes. Pneumonia showed the lowest performance, with an internal test AUROC of 0.749, 11% larger than the corresponding Baseline model. A similar AUROC was observed in external validation, but was 42% larger than the Baseline’s. The model designed for wheezing disorders was much more performant with an AUROC of 0.912, 2% larger than the Baseline’s. However, this model was much less performant in external validation, with an AUROC of 0.743, 13% larger than the Baseline’s. Finally, the bronchiolitis submodel had an internal test AUROC of 0.939, 1% lower than the Baseline’s. On external validation, the model had a similar AUROC of 0.870, 3% lower than the Baseline’s, with a redistribution of sensitivity and specificity compared to internal validation. The specificity vs sensitivity trade-off could be specified a priori, but was not performed in this work.

**Table 1:** Performance breakdown of binary models: Internal CV results.

Target	Model	Sensitivity (SD)	Specificity (SD)	AUROC (SD)
**Control**	Baseline	0.501 (0.033)	0.938 (0.017)	0.879 (0.032)
	*DeepBreath*	0.856 (0.053)	0.847 (0.039)	0.931 (0.014)
**Pneumonia**	Baseline	0.000 (0.000)	0.996 (0.011)	0.637 (0.044)
	*DeepBreath*	0.508 (0.197)	0.858 (0.046)	0.749 (0.097)
**Wheezing Disorder**	Baseline	0.426 (0.065)	0.938 (0.015)	0.889 (0.038)
	*DeepBreath*	0.792 (0.088)	0.880 (0.036)	0.912 (0.033)
**Bronchiolitis**	Baseline	0.761 (0.102)	0.900 (0.018)	0.949 (0.018)
	*DeepBreath*	0.830 (0.077)	0.880 (0.044)	0.939 (0.021)

**Table 2:** Performance breakdown of binary models: External validation.

Target	Model	Sensitivity (CI95)	Specificity (CI95)	AUROC (CI95)
**Control**	Baseline	0.241 (0.139–0.372)	0.830 (0.742–0.898)	0.703 (0.622–0.785)
	*DeepBreath*	0.770 (0.645–0.868)	0.840 (0.756–0.904)	0.887 (0.839–0.936)
**Pneumonia**	Baseline	0.000 (0.000–0.079)	1.000 (0.968–1.000)	0.315 (0.226–0.405)
	*DeepBreath*	0.469 (0.325–0.617)	0.839 (0.760–0.900)	0.739 (0.656–0.821)
**Wheezing Disorder**	Baseline	0.419 (0.245–0.609)	0.693 (0.605–0.772)	0.614 (0.513–0.715)
	*DeepBreath*	0.636 (0.451–0.796)	0.701 (0.616–0.777)	0.743 (0.651–0.836)
**Bronchiolitis**	Baseline	0.542 (0.328–0.744)	0.933 (0.876–0.969)	0.896 (0.843–0.949)
	*DeepBreath*	0.500 (0.291–0.709)	0.923 (0.867–0.961)	0.870 (0.796–0.943)

Again, each binary model classifies itself vs all other classes. In Supplementary Table 4, we stratify these performance metrics by class to show the sensitivity of that model for correctly classifying each ‘other’ class as ‘other’. The results show that all binary models tend to misclassify Pneumonia the most of all sub-classes.

### *DeepBreath*—combining binary classifiers

**Fig. 2:**
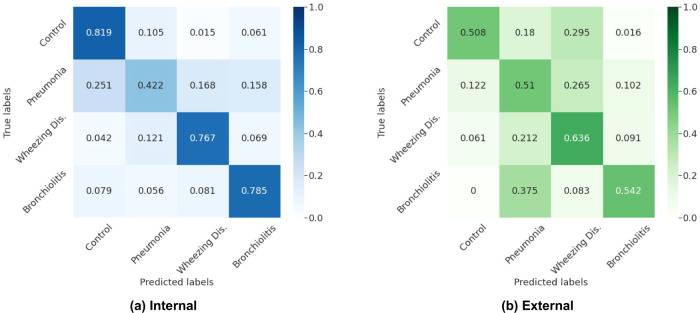
*DeepBreath* confusion matrices for multi-class predictions. A confusion matrix was computed for every CV model on the corresponding test fold. **a** The internal confusion matrix was then obtained by taking the average of these intermediate confusion matrices. **b** The external confusion matrix is computed on the aggregated patient predictions (ensemble output). The rows are normalized to add up to 1.

We combine the *DeepBreath* submodels for multi-class classification using the intermediate outputs of the models, which are concatenated and normalized, before given as input to a multinomial logistic regression. The confusion matrices for the internal test folds and the external validation data are depicted in Fig. 2. On the internal test folds, the classification performance of the joint model shows only a minimal reduction in class-recalls (sensitivity). For instance the combined model reports an internal sensitivity of 0.819, 0.422, 0.767 and 0.785 for control, pneumonia, wheezing disorders, and bronchiolitis respectively. This represents a decrease in performance of less than 5% for all but pneumonia that decreases by 8.6%. On external validation, pneumonia and bronchiolitis improve by about 4%, wheezing disorders stays the same, and control drops significantly. However, as the discrimination of healthy vs pathological patients is not as clinically relevant (patients with no respiratory symptoms do not have an indication for lung auscultation) it can be argued that low sensitivity in this class is not a significant issue.

### Attention values: interpretation

Supplementary Fig. 3 shows the distribution of **MAD** values of the Geneva recordings, w.r.t. the patient diagnoses. For control patients, the distribution seems to be symmetric about the origin, showing that the model doesn’t pay more attention to either of the respiration phases. Interestingly, the distribution is right-skewed for most of the respiratory diseases. This would indicate that the CNN model focuses more on expiration than inspiration. This could be explained by the fact that adventitious sounds more commonly appear during the expiration phase.

**Fig. 3:**
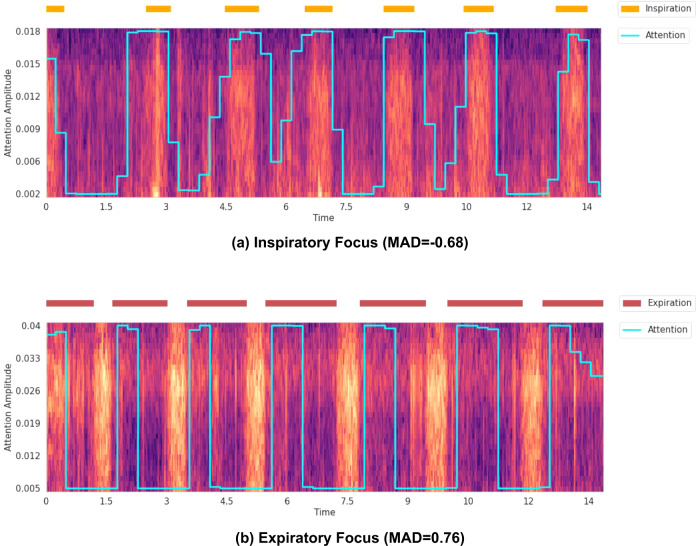
Example attention curves, returned by the CNN classifier that discriminates healthy recordings from pathological ones. The attention curves are overlaid on the recording spectrograms, which are given as inputs to the CNN classifier. Depending on the MAD value, either inspiration or expiration phases are provided as a reference. The respiration phases were extracted from the recording annotations. **a** The recording has a negative MAD, and its attention curve is shown with the inspiration phases. **b** The recording has a positive MAD, and its attention curve is shown with the expiration phases.

Figure 3 illustrates examples of segment-attention curves, overlaid on the corresponding spectrograms that were given as input to the CNN classifier. Depending on the **MAD** value, inspiration or expiration signals are also shown, which can be extracted from the recording annotations. By definition, a recording has a low **MAD** if the model focuses more on inspiration than expiration segments (as labelled by the annotators). The lower the **MAD** value, the more peaks of the attention curve should align with the extracted inspiration phases. Conversely, for a high **MAD**, the model focuses more on expiration segments, and the attention curve peaks should align with the extracted expiration phases. Two examples with high magnitude **MAD** values are shown. The first recording comes from an asthma patient and has a negative **MAD**, the alignment with the inspiration segments indicates that the model focuses on the inspiration phases of the patient. Asthma wheezing is known to have biphasic wheezing (especially with increasing severity), and while expiratory wheezing may be more obvious to the human ear, the singularity of inspiratory wheezing may thus serve as a distinguishing characteristic identified by the model^22^.

The second example is from a patient with obstructive bronchitis. The recording’s **MAD** is positive, focusing its attention on the expiration phases, exclusively at the beginning of the expiration. This expiratory wheezing also aligns with clinical expectation of the disease.

### Inference optimization

**Fig. 4:**
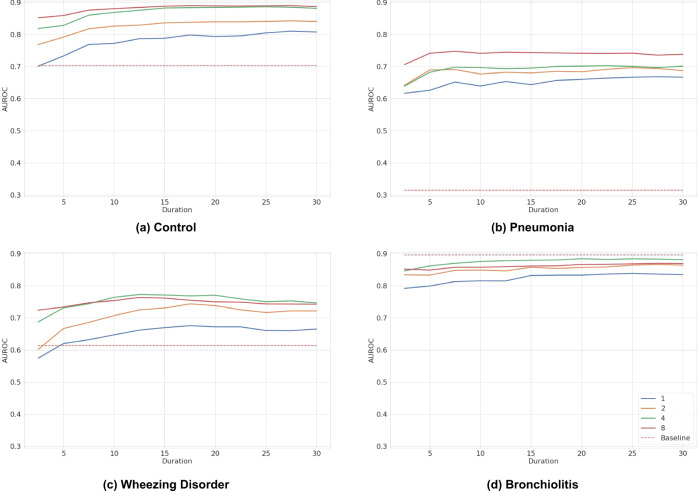
Optimal duration and combination of inference audio. Each graph represents a trained binary model for **a** Control, **b** Pneumonia, **c** Wheezing Disorders and **d** Bronchiolitis. Each solid line is the AUROC performance resulting from the external validation data comprising one of four combinations of anatomical positions (1, 2, 4 and 8). The AUROC is plotted over the duration of the test set samples in seconds ranging from 2.5 to 30. The dashed lines show the performances of the clinical baseline models that only use age and respiratory rate.

As *DeepBreath* can make inference on a variable duration and combination of audio recordings, we seek to determine the minimum length and smallest combination of recordings that the trained *DeepBreath* model requires to ensure its reported performance. Figure 4 shows the performance of the binary *DeepBreath* models on variable lengths and anatomical combinations of auscultation audio recordings. Only samples with at least 30 seconds and 8 positions available are used, resulting in a slight deviation from the above reported results. For each binary model, we explore the minimal number of anatomical positions (all 8, a combination of 4, 2 or a single recording). Each sample is then cropped to various durations (from 2.5 seconds to 30 seconds) and the AUROC of each combination and duration is plotted. Comparing results to the baseline using all 8 positions with the full 30 second duration (i.e. the red line at the 30-second mark on each graph). We see that the performance is matched when using recordings from a combination of just 4 positions. An exception is pneumonia which requires all 8 positions to avoid the 0.04 loss in AUROC. All position combinations also maintain their performance until their durations are shortened to around 10-seconds.

Thus, *DeepBreath* maintains performance when given just 10 seconds of auscultation recordings from 4 positions.

## Discussion

In this study, we evaluate the potential of deep learning to detect the diagnostic signatures of several pediatric respiratory conditions from lung auscultation audio. Particular attention was dedicated to the identification and correction of hidden biases in the data. We standardise acquisition practices and perform robust model evaluation as well as explicitly report external validation on independently collected cohorts with a broad geographic representation.

Despite these strict constraints, *DeepBreath* returned promising results, discriminating several disease classes with over 90% area under the receiver operating characteristic (AUROC) curve, and often significantly outperforming a corresponding Baseline model based on age and respiratory rate (RR). The results on the external validation were, on average, less than 8% lower, compared to 21% lower for the Baseline models. Even the binary classifier for pneumonia achieved 75 and 74% AUROC in internal and external validation respectively. This value is considered surprisingly good in light of the notorious lack of international gold standard for the diagnosis of the disease and the international context of our cohort. *DeepBreath* also showed potential to maintain stable performance under sparse data conditions. Indeed, using just 5-10-second recordings from 4 anatomic positions on the thorax was equivalent to using 30 second clips from all 8 positions.

The interpretable-by-design approach of attention-spectogram mapping further validates the clinical relevance of our results, offering unique insights into the model’s decision-making process. These tangible visualizations map the predictive *attention* on the audio spectrogram allowing it to be aligned with human annotations of the respiration cycle. We found intuitive concordance with inspirations and expirations, which better ensures that discrimination is based on respiratory signals rather than spurious noise. Thus, *DeepBreath* can be interrogated by medical experts, allowing them to make an informed assessment of the plausibility of the model’s output. This interpretation technique could possibly be used as a more objective way to identify adventitious sounds, requiring no (laborious) time-stamped labeling of audio segments, but using only clip-level annotations. Extrapolating the possible applications of this approach, it could even provide a method for standardising the unique acoustic characteristics of respiratory disease into a visually interpretable set of patterns that could find a use in medical training.

As it is preferable (albeit more challenging) to have a single model able to discriminate a range of differential diagnoses, we combined all the binary classifiers into a single multi-class diagnostic classifier. While this showed very promising results on our internal test folds, its performance was significantly reduced in external validation. This is a strong indication that more data is needed before such an architecture would be ready for a real-world deployment. The performance was likely particularly limited by the important data imbalance regarding diagnosis and recording centre. Another limitation of this work, is that *DeepBreath* was trained and evaluated on a data set generated with a single stethoscope brand. Future work to render the models device-agnostic would be valuable to expanding their potential scope of deployment.

Taken together, the *DeepBreath* model shows the robust, promising and realistic predictive potential of deep learning on lung auscultation audio; and offers a framework to provide interpretable insights of the objective audio signatures for one of the most frequently misinterpreted clinical exams.

## Methods

### Participants and cohort description

A detailed breakdown of participants stratified by geographic site and diagnostic label is provided in Table 3. A total of 572 patients were recruited in the context of a multi-site observational cohort study using a standardised acquisition protocol. Selection criteria aimed to recruit patients below the age of 16 presenting at paediatric outpatient facilities who had suspected lower respiratory tract infection, acute asthma or obstructive bronchitis. Patients with known chronic underlying respiratory disease (e.g. fibrosis) or heart failure were excluded. In total, 71% (*n* = 407/572) were clinically diagnosed *cases* with one of three diagnostic labels: (i) pneumonia, (ii) wheezing disorders, or (iii) bronchiolitis. The remaining 29% (*n* = 165/572) were age- and sex-balanced *controls* with no respiratory symptoms, consulting at the same emergency unit for other complaints. Distributions of age and respiratory rates between cases and controls are provided in Supplementary Fig. 2.

**Table 3:** Number of patients used to train the models in this study, stratified by diagnosis and collection site.

	Control	Pneumonia	Wheeze^a^	Bronchiolitis	Total *n*
GVA	23	27	66	4	120
POA	80	38	21	91	283^b^
DKR	17	5	8	1	31
MAR / RBA	32	0	19	8	59
YAO	13	44	6	16	79
Total *n*	165	114	120	120	572
(%)	(29)	(20)	(21)	(21)	(100)

*GVA* Geneva, Switzerland, *POA* Porto Alegre, Brazil, *DKR* Dakar, Senegal, *MAR* Marrakesh, Morocco, *RBA* Rabat, Morocco, *YAO* Yaound, Cameroon, Total *n* total number of patients, stratified by diagnosis.

^a^Wheezing Disorder comprises obstructive bronchitis (30%) and asthma (70%).

^b^In POA, 53 cases had no differentiation between bronchiolitis and wheezing disorder and are not listed in a specific column.

All participants were recruited between 2016 and 2020 at the outpatient departments of six hospitals across five countries (120 in Geneva, Switzerland; 283 in Porto Alegre, Brazil; 31 in Dakar, Senegal; 79 in Yaoundé, Cameroon; and finally 59 from Rabat and Marrakesh in Morocco). Detailed summary statistics are provided in Supplementary Table 2. All diagnoses are validated by two medical doctors. Pneumonia is diagnosed radiologically where possible or by the presence of audible crackles and/or febrile respiratory distress. 90% of the pneumonia cases were reported as bacterial, and the rest as viral. These are grouped into a single category of *pneumonia* due to the notorious difficulty of distinguishing between them objectively. Bronchiolitis and wheezing disorders (comprising asthma and obstructive bronchitis) are diagnosed clinically.

### Ethics

The study is approved by the Research Ethics Committee of Geneva and local research ethics boards in each participating country. All patient’s caregivers provided written informed consent.

### Dataset acquisition

A series of digital lung auscultation (DLA) audios were acquired from each of the 572 recruited patients across eight anatomic sites (one in each quadrant of the anterior and posterior thorax). DLAs had an average duration of 28.4 seconds (range 1.9-30). Only 2.8% of patients (*n* = 16/572) did not have all eight recordings present. Collectively, 4552 audio recordings covered 35.9 hours of breath sounds. All recordings were acquired on presentation, prior to any medical intervention (e.g. bronchodilators or supplemental oxygen). DLAs were recorded in WAVE (.wav) format with a Littmann 3200 electronic stethoscope (3M Health Care, St. Paul, USA) using the Littmann StethAssist proprietary software v.1.3 and *Bell Filter* option. The stethoscope has a sampling rate of 4,000 Hz and a bit depth of 16 bits.

### Dataset description

Recordings have one of four clinical diagnostic categories: control (healthy), pneumonia, wheezing disorders (asthma and bronchitis pooled together) and bronchiolitis. The diagnosis was made by an experienced pediatrician after auscultation and was based on all available clinical and paraclinical information, such as chest X-ray when clinically indicated. For the purposes of this study, obstructive bronchitis and asthma are grouped under the label “wheezing disorder” due to their similar audible wheezing sound profile and treatment requirements (bronchodilators).

Rather than having a single model with four output classes (listed above), we adopted a “one-versus-rest” approach where four separate binary classification models discriminate between samples of one class and samples of the remaining three classes. The combination of the outputs of the resulting ensemble of four models yields a multi-class output. Such an approach allows for separating the learned features per class, which in turn improves the interpretability of the overall model. Moreover, such a composite model more easily permits a sampling strategy that compensates for the imbalanced class distribution in the training set. For a fair comparison, an identical model architecture was used for all the diagnoses in this study. For a broader classification of pathological breath sounds, predictions from the three pathological classes are grouped into a composite “pathological” label.

Several clinical variables were collected for each patient. Age and respiratory rate (RR) are used in a clinical baseline model described below. Age was present for all patients. RR was recorded by clinical observation at the bedside and is present for 94% (*n* = 536/572) of the patients. The baseline is thus computed on this subset.

Recordings from the Geneva site (cases and controls) were annotated by medical doctors to provide time-stamped inspiration and expiration phases of the breath cycle. These will serve as a reference for the interpretation methods described below.

### Data partitioning (train:tune:test split and external validation)

Considering potential biases in the data collection process, and a model’s tendency to overfit in the presence of site-specific background noise, it is particularly important to ensure balanced representation in data splits and even more critical to explicitly report results on an external validation set from an independent clinical setting. As the Geneva (GVA) and Porto Alegre (POA) recordings were the most abundant and diverse in terms of label representation, they were thus used for training, internal validation (i.e. tuning) and testing.

To ensure that performance was not dependent on fortuitous data partitioning, nested 5-fold stratified cross validation (CV) was performed to obtain a distribution of performance estimates which are then reported as a mean with standard deviation (SD). The random fold compositions are restricted to maintain class balance (i.e. preserving the percentage of samples for each class).

The other centres of Dakar (DKR), Marrakesh (MAR), Rabat (RBA) and Yaoundé (YAO) were used for external validation, i.e. the trained model has never seen recordings coming from these centres, and the recordings have not been used for hyper-parameter selection. For external validation, the models trained on internal data using nested CV can be seen as an ensemble. Model predictions were averaged across the ensemble, to get a single prediction per patient. Performance metrics were then computed over the external validation set and are reported along with 95% confidence intervals (CI95%).

**Fig. 5:**
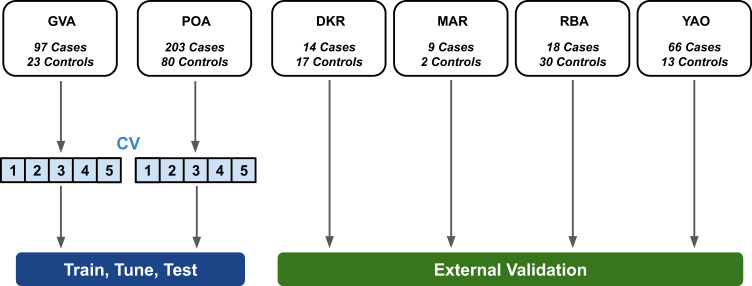
Dataset partitioning strategy. Geneva (GVA) and Porto Alegre (POA) are used for training, internal validation (tuning), model selection and testing. External validation is performed on independently collected recordings from Dakar (DKR), Marrakesh (MAR), Rabat (RBA) and Yaoundé (YAO). CV Cross Validation.

The partitioning strategy is illustrated in Fig. 5.

### Clinical baseline

To assess the clinical value of using *DeepBreath* as opposed to a simpler model based on basic clinical data, we trained a set of equivalent baseline models using logistic regression on age and respiration rate. These two features are relatively simple to collect and are routinely used to assess patients with respiratory illness. For instance, in our dataset, the age of bronchiolitis patients is significantly lower than for the other disease groups, and the RR of all disease groups differ significantly between each other (see Supplementary Table 2). This suggests that these features are likely predictive for the diseases, provided that their distributions are reasonably linearly separable.

The same train:tune:test splits as for the training of *DeepBreath* were used for the baseline. In a first step, the train and tune folds of the nested CV were used to obtain the best hyper-parameters using grid-search. The best hyper-parameters found for each disease classifier are reported in Supplementary Table 3. Then, 20 models corresponding to the different combinations of train folds were trained for each disease using the best hyper-parameters. The same evaluation procedure as for *DeepBreath* was used (described in Statistical methods).

### *DeepBreath* : model design

**Fig. 6:**
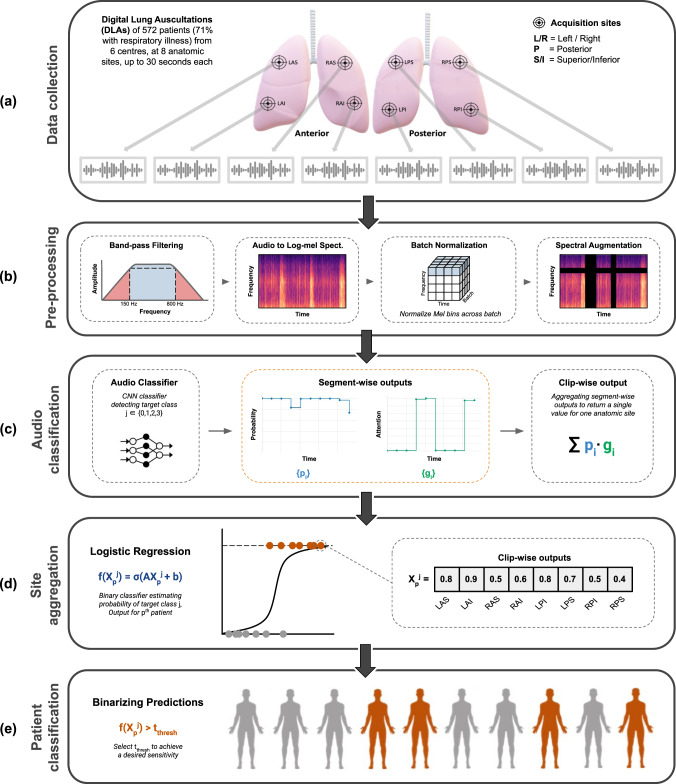
Overview of the *DeepBreath* binary classification model. This binary classification architecture is trained for each of the four diagnostic classes. Top to bottom: **a** Data collection. Every patient has 8 lung audio recordings acquired at the indicated anatomical sites. **b** Pre-processing. A band-pass filter is applied to clips before transformation to log-mel spectograms which are batch-normalized and augmented and then fed into an (**c**) Audio classifier. Here, a CNN outputs both a segment-level prediction and attention values which are aggregated into a single clip-wise output for each site. These are then (**d**) Aggregated by concatenation to obtain a feature vector of size 8 for every patient, which is evaluated by a logistic regression. Finally (**e**) Patient-level classification is performed by thresholding to get a binary output. The segment-wise outputs of the audio classifier are extracted for further analysis. Note that the way the 5-second frames are created during training is not shown here (zero padding or random start).

*DeepBreath* is a composite binary classifier trained separately for each diagnostic category (e.g. pneumonia vs not pneumonia etc.). It consists of a CNN audio classifier that produces predictions for single recordings, followed by a logistic regression that aggregates the predictions of the recordings corresponding to the eight anatomical sites. It then outputs a single value per patient. Intermediate outputs, such as the segment-wise predictions for a recording, are extracted for later interpretability analysis. Figure 6 depicts the pipeline of the *DeepBreath* binary classifier.

### Preprocessing: generating fixed-size inputs (only during training)

The training phase requires fixed-size inputs for batch-wise processing. Here, 5-second audio frames are presented to the classification architecture. Shorter recordings are zero-padded. For longer recordings, we extract a 5-second frame from a random start within the recording. The resting respiratory rhythm for an adult is around two seconds inhalation and three seconds exhalation^23^. For a child, a full respiration cycle is shorter, and respiratory disease tends to reduce this further. Thus, a 5 second crop would generally ensure at least one full respiration cycle irrespective of the random starting position. At inference time, the model can process the entire recording, no matter the duration. There is thus no need for zero-padding.

### Preprocessing: spectral transformation

DLAs are first converted to log-mel spectrograms using *torchaudio*. The spectrograms are generated by computing discrete Fourier transforms (DFT) over short overlapping windows. A Hann window length of 256 samples and a hop length of 64 samples were used. At a 4000 Hz sampling rate, this corresponds to a window duration of 64 ms, and a hop duration of 16 ms. This process is known as the Short Time Fourier Transform (STFT). With a hop duration of 16 ms, we get 62.5 frames per second (rounded up in practice). To get log-mel spectrograms, the obtained magnitude spectra for frequencies between 250 and 750 Hz are projected onto 32 mel-bands, and converted to logarithmic magnitudes. Again, a narrow frequency range was chosen to reduce the interference of background noises. The log-mel spectrogram of a 5-second clip has a shape of 32 × 313. Before being processed by the CNN model, the log-mel spectrograms are normalized with Batch Normalization^24^. Because for spectrograms, the vertical translational invariance property does not hold (unlike standard images, spectrograms are structured and contain different information in the different frequency bands), each frequency band is normalized independently of the others. During training, SpecAugment^25^ is performed. This augmentation technique, that was initially developed for speech processing, masks out randomly selected frequency and time bands.

### Preprocessing: audio-level predicitons

We adapted an architecture from the PANN paper^4^ codebase. The original *Cnn14_DecisionLevelAtt* model was designed for sound event detection. Our model consists of 5 convolutional blocks. Each convolutional block consists of 2 convolutional layers with a kernel size of 3 × 3. Batch normalization is applied between each convolutional layer to speed up and stabilize the training. After each convolutional layer and Batch normalization, we use ReLU nonlinearity^26^. We apply average pooling of size 2 × 2 after the first 4 convolutional blocks for down-sampling, as 2 × 2 average pooling has been shown to outperform 2 × 2 max pooling^27^. After the last convolutional layer, the frequency dimension is reduced with average pooling. Average and max pooling of size 3 and stride 1 are applied over the time dimension and summed to get smoothed feature maps. Then, a fully connected layer (with in_features = 1024) followed by a ReLU nonlinearity is applied to each of the time-domain feature vectors (of size 1024, which corresponds to the number of channels of the last convolutional layer).

Let *x* = {*x*_1_, …, *x*_*T*_} be the sequence of feature vectors obtained from the previous step. Here *T* is the number of segments, which depends on the duration of the recording and the number of pooling operations applied after the convolutional blocks. In order to get segment-level predictions, an attention block is applied to the feature vectors. This attention block outputs two values per feature vector, by applying two distinct fully connected layers to each of the feature vectors (implemented with Conv1d in PyTorch). The first fully connected layer is followed by a sigmoid activation function and outputs a value *p*(*x*_*i*_). The second fully connected layer is followed by a Tanh nonlinearity and outputs a weight *v*(*x*_*i*_). These weights are then normalized over all segments with a softmax function:

1 $$g({x}_{i})=\frac{exp(v({x}_{i}))}{\mathop{\sum }\nolimits_{j = 1}^{T}exp(v({x}_{j}))},i=1,\ldots ,T\,.$$

The value *g*(*x*_*i*_) is called the attention value of the *i*^*th*^ segment. It controls how much the prediction *p*(*x*_*i*_) should be attended in the clip-level prediction. The clip-level prediction is now obtained as follows:

2 $$p(x)=\mathop{\sum }\limits_{i=1}^{T}g({x}_{i})p({x}_{i})\,,\qquad \,{{\mbox{thus:}}}\,\quad p(x)\in [0,1]\,.$$

Dropout^28^ is applied after each downsampling operation and fully connected layers to prevent the model from overfitting.

### Preprocessing: patient-level prediction (site aggregation)

To obtain patient-level predictions, we combine the predictions of each of the 8 anatomic sites of a single patient and concatenate them to obtain a vector of size 8. In this step, only patients for which all eight recordings are available were selected. This new set of features can be used to construct new datasets for the train, tune and test folds. We chose to fit a logistic regression model to these new features, because it has properties pertinent to our setting, such as being interpretable and returning the probability of an event occurring. With this model, there is no need for a tune set to save the best model during training. Thus we concatenated the feature matrices of the train and tune folds, to obtain a new feature matrix that will be used to fit the logistic regression model. The test features will be used to evaluate our model. Note that this is the first time that the test data is used. The CNN model is not trained during this second phase and never sees the test data.

### Combining binary classifiers

**Fig. 7:**
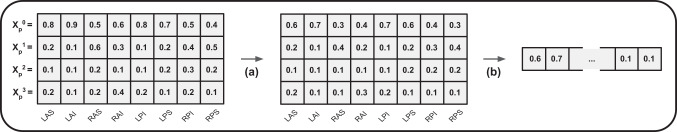
Feature construction for multi-class classification. For every patient, the four binary models produce a feature vector of size 8, corresponding to the predictions of the recordings from the 8 anatomical sites. Those feature vectors are concatenated to form a prediction array of size 4 (classes) × 8 (sites). Then, the following operations are applied to the prediction array: (**a**) Column normalization of the prediction array (**b**) Flattening to obtain a feature vector of size 32. The final feature vector is than given as input to the multinomial logistic regression.

For diagnostic classification, we combine the positional feature vectors (corresponding to the eight anatomical sites) of the four CNN audio classifiers. The feature vectors are concatenated to form a prediction array of size 4 × 8, and the columns are then L1-normalized (for each column, the sum of its values is equal to 1). The idea behind this normalization is that recordings—which were identified by multiple CNN models as having a high likelihood of belonging to their respective class—should matter less in the aggregated patient prediction. Similar to the aggregation step in the binary models, we obtain new feature datasets for the training, tune and test folds. Figure 7 shows how that data is generated. A multinomial logistic regression is then trained to predict a patient’s diagnosis. Again, the test features are only used to evaluate the model.

### Extracting attention values

*DeepBreath* is interpretable by design. At the level of the CNN model, we can plot the segment-level predictions ${\{p({x}_{i})\}}_{i = 1}^{T}$, and the attention values ${\{g({x}_{i})\}}_{i = 1}^{T}$. These values can identify the parts of the recording that are most deterministic for the prediction over the time dimension. Comparing these values to segment-level annotations made by medical doctors (identifying inspiration and expiration), we can visualize how the model interprets disease over the breath cycle and thus allow clinicians to interrogate the model’s alignment to physiology.

Every CNN audio classifier passes through a recording and computes segment-level outputs, before aggregating those intermediate outputs to return a single clip-level prediction. The duration captured by a single segment is determined by the size of the receptive field of the CNN architecture. The receptive field of the final convolutional layer has a width of 78, which corresponds to a duration of 1296 ms. For every segment, the CNN model computes an attention value *g*(*x*_*i*_) and a prediction *p*(*x*_*i*_). The attention value *g*(*x*_*i*_) determines how much the segment prediction *p*(*x*_*i*_) is attended in the overall clip-level output *p*(*x*). Plotting ${\{g({x}_{i})\}}_{i = 1}^{T}$ allows us to identify parts of the recording (hence the respiration) that have a high contribution to the clip-level prediction. In order to interpret these singled-out parts, we made use of annotations of breath sounds, that were provided for the recordings from Geneva. With those annotations we can evaluate whether there is a similarity between the way medical experts label breath sounds, and the way respiration is perceived by a model (that was trained for diagnosis prediction without any knowledge of respiration phases or sounds).

### Mean-Attention Difference (MAD)

To better quantify how attention correlates to human annotations of the breath cycle, we take the healthy vs pathological binary classifier *DeepBreath* submodel as an example and define a metric called the Mean-Attention Difference (**MAD**). Given a recording, the CNN model generates the segment-attention values ${\{g({x}_{i})\}}_{i = 1}^{T}$. Let ${\left\{g({x}_{{u}_{k}})\right\}}_{k = 1}^{{T}_{in}}$ and ${\left\{g({x}_{{v}_{l}})\right\}}_{l = 1}^{{T}_{out}}$ be the sub-sequences of segment-attention values corresponding to inspiration and expiration phases, respectively. Those sub-sequences can be identified with the segment-level annotations that provide timing information. We define

3 $${\alpha }_{in}=\frac{1}{{T}_{in}}\left(\mathop{\sum }\limits_{k=1}^{{T}_{in}}g({x}_{{u}_{k}})\right)$$

4 $${\alpha }_{out}=\frac{1}{{T}_{out}}\left(\mathop{\sum }\limits_{l=1}^{{T}_{out}}g({x}_{{v}_{l}})\right)$$

as the mean attention values of the inspiration and expiration phases, respectively. The **MAD** is now defined as follows:

5 $${{{\bf{MAD}}}}=\frac{{\alpha }_{out}-{\alpha }_{in}}{\max ({\alpha }_{out},{\alpha }_{in})}$$

Note that when **MAD** > 0, it means that the model focuses more on expiration segments than inspiration segments. The opposite is true when **MAD** < 0. If our model gives equal importance to both inspiration and expiration, then a plot of **MAD** values across all recordings should show a symmetric distribution around the origin.

### Model training

The CNN classifiers were trained with a Binary Cross Entropy (BCE) loss with clip-level predictions and labels. A batch-size of 64, and an AdamW optimizer^29^, combined with a 1cycle learning rate policy^30^, were used. The 1cycle policy increases the learning rate (LR) from an initial value to some maximum LR and then decreases it from that maximum to some minimum value, much lower than the initial LR. The maximum LR was set to 0.001. The default parameters of the PyTorch implementation were used otherwise. The weight decay coefficient of the AdamW optimizer was set to 0.005.

A balanced sampling strategy was implemented to address the class imbalance, w.r.t. the diagnoses and recording centres. First, when training the model to identify one of the four categories, it is important that the three other categories are equally represented. This is because otherwise, the model might learn to distinguish between two categories, which is easier. Second, sampling recordings from different locations in a balanced way is also important. Our experiments have shown that the model can learn to distinguish recording locations with relatively high accuracy. Thus, there is a risk that the model may learn spurious features when training it to distinguish pathologies by focusing on centre-specific background noise for over-represented categories in one location. Hence, our sampling strategy aims to enforce that the CNN learns location-invariant representations, as much as possible, as some localized features might yet be desired. With a balanced sampling, an epoch corresponds to a full sweep of the data. The number of batches per epoch is set such that *N* recordings are sampled during an epoch, where *N* is the total number of recordings. With over-sampling (or under-sampling), we are bound to have duplicates over an epoch (or missed recordings, respectively).

Every model was trained for 100 epochs. After 60 epochs, the model performance is evaluated on the tune fold, to save the best model w.r.t. mean positional AUROC, obtained as follows. For every recording in the tune fold, the CNN classifier predicts a score in [0,1]. Those scores are then split into eight different groups, depending on the position that was used for the recording. For every position, AUROC is computed on the positional scores, and then the mean of these eight AUROC values is returned. The idea behind this metric is that the model might perform differently depending on the position (more confident for certain positions than for others), and since we are feeding the positional scores to another model (logistic regression), metrics that require us to binarize the scores like accuracy or F1-score might not be adapted to the task.

### Inference optimization

As *DeepBreath* can perform inference on variable lengths and anatomical positions of audio, we determine the minimum length and smallest combination of recordings that the trained *DeepBreath* model requires to ensure its reported performance. These experiments are performed exclusively on the test set.

We compare the performance of using all 8 sites to a combination of 4 or 2 sites or a single site. The combinations are selected from the anterior superior (left and right) recordings, which are known to have less audible interference from the heart and stomach.

To then explore the minimum duration required for each combination, we create variable duration crops, ranging between 2.5 and 30 seconds in 2.5 second increments.

### Statistical methods

For every CV iteration, a model was trained using the internal train and tune folds. Sensitivity, specificity and area under the receiver-operator-characteristic curve (AUROC) values were computed on the corresponding test fold. With 5-fold nested CV, this procedure was repeated 20 times. The internal performance metrics are reported as a mean, together with SD, taken over the CV iterations. Each of the 20 trained models was also evaluated on the entire external validation set. For every patient, predictions were then averaged across this ensemble of models, to obtain a single aggregated prediction. Sensitivity, specificity and AUROC values were computed on the aggregated predictions. To assess the variability of the performance estimates, we provide 95% Clopper-Pearson confidence intervals^31^ for sensitivity and specificity, and 95% DeLong confidence intervals for AUROC^32^.

### Reporting summary

Further information on research design is available in the Nature Research Reporting Summary linked to this article.

## Supplementary information

Supplementary Material

Reporting Summary

## Data Availability

Anonymized data are available upon reasonable request (alain.gervaix@hcuge.ch) which matches the intention to improve the diagnosis of paediatric respiratory disease in resource-limited settings. The audio used in the study are not publicly available to protect participant privacy. Unlimited further use is not permissible from the informed consent.

## Code availability

The full code and test sets are available at the following GitHub repository: https://github.com/epfl-iglobalhealth/DeepBreath-NatMed23. The repository also provides instructions on how to access and train this model on DISCO, our web-based platform for distributed collaborative learning https://epfml.github.io/disco/#/ to provide access to privacy-preserving federated and decentralised collaborative training and inference.
