# Supplementary material for: DeepBreath—automated detection of respiratory pathology from lung auscultation in 572 pediatric outpatients across 5 countries
Source: NPJ Digit Med. 2023 Jun 2;6:104. doi: 10.1038/s41746-023-00838-3 (PMC10238513; doi:10.1038/s41746-023-00838-3)
Supplement: Supplementary file 1 — Supplementary Material [file 41746_2023_838_MOESM1_ESM.pdf]

Supplementary information of:
DeepBreath—Automated Detection of Respiratory Pathology from Lung Auscultation in 572 Pediatric Outpatients across 5 Countries

Supplementary figures

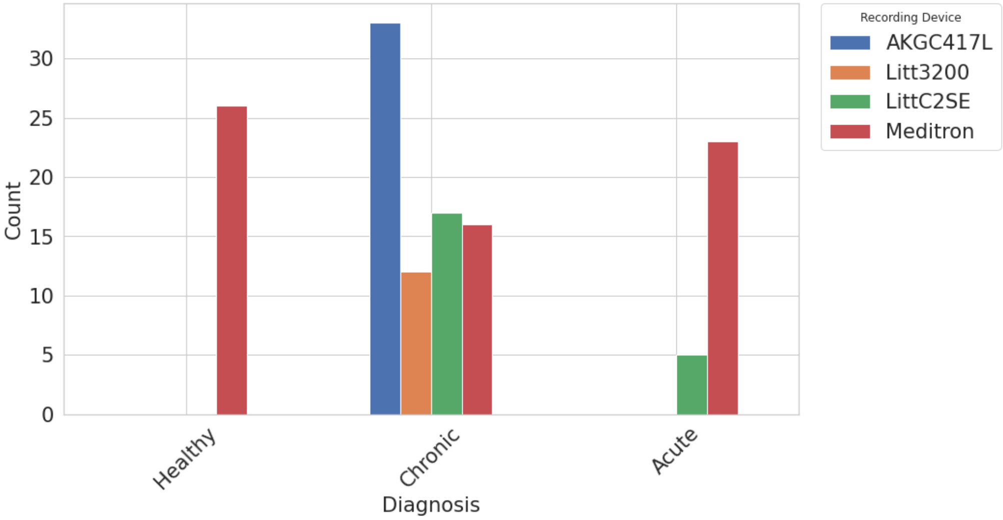

Supplementary Fig. 1. Biased distribution of recording devices per diagnostic category in the public ICBHI (International Conference on Biomedical Health Informatics) data set [1]. A deep learning model could simply use the audible signature of the recording device to discriminate classes resulting in clinically irrelevant models with seemingly high performance estimates.

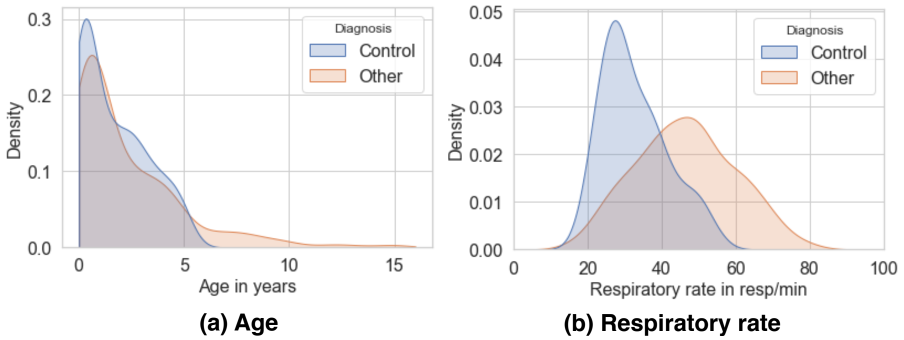

Supplementary Fig. 2. Age and respiratory rate distributions of internal dataset's cohort.
In both panels, the two histogram distributions were smoothed with a Gaussian kernel with bandwidth parameter equal to 1, and were further normalized to ease their visual comparison. **(a) Age distribution.** The means of the Control and Other groups are respectively 1.6 and 2.2 years. **(b) Respiratory rate distribution.** The means of the Control and Other groups are respectively 32.7 and 46.4 respirations per minute.

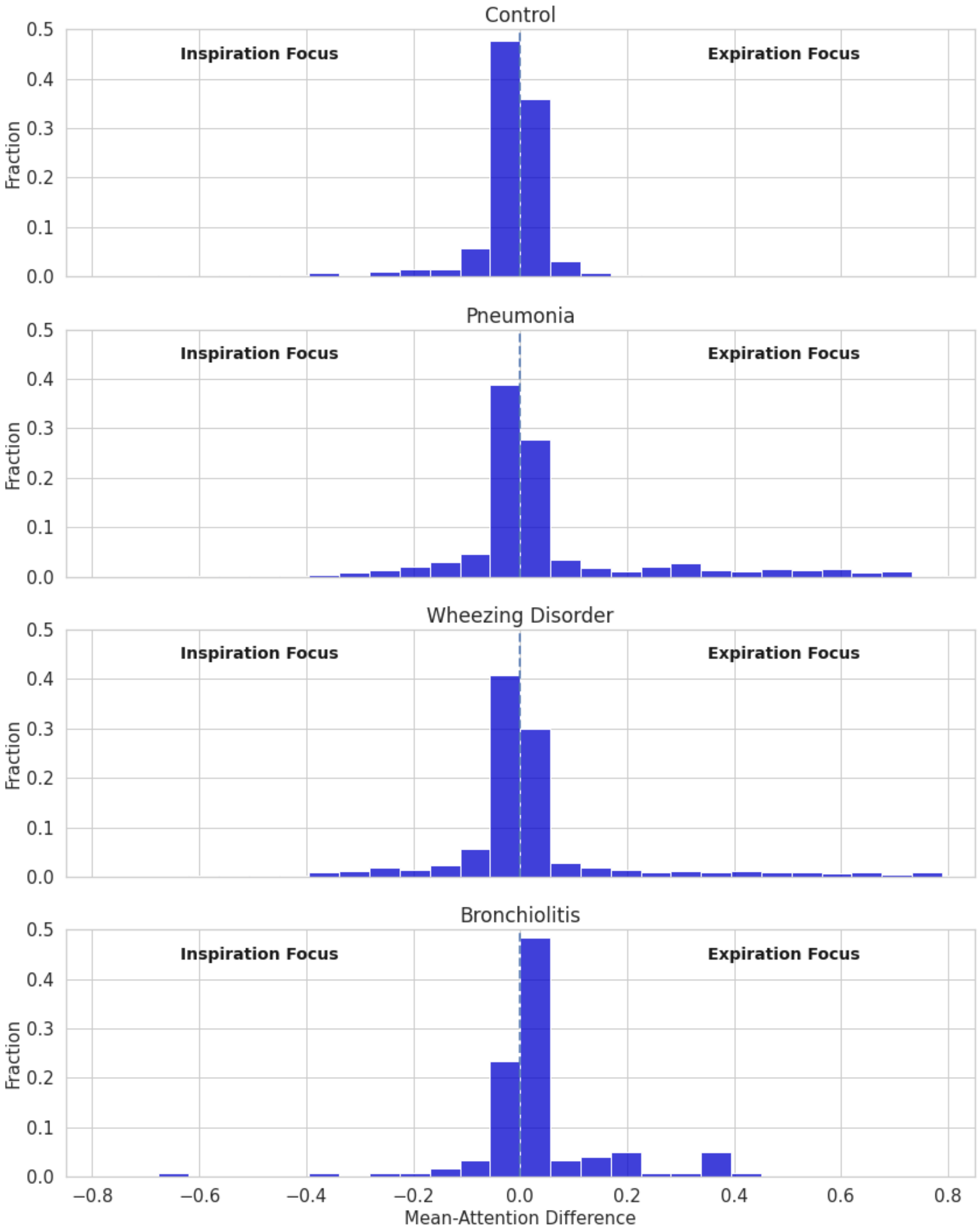

Supplementary Fig. 3. Distribution of recording MAD values, produced by the *DeepBreath* submodel for healthy vs pathological classification. Distributions are shown per type of diagnosis, to highlight variations in the way the model pays attention to different respiration phases, depending on the underlying pathology.

Supplementary tables

Supplementary Table 1. Biased distribution of age per diagnostic category in the public ICBHI (International Conference on Biomedical Health Informatics) data set [1]. A deep learning model could simply use the age-related differences in respiratory rate to discriminate classes resulting in clinically irrelevant models with seemingly high performance estimates.

	Healthy	Chronic conditions	Acute conditions
Age, yrs (mean)	6.2	67.2	15.9
CI95%	*4.1—8.3*	*64.4—69.9*	*5.0—26.7*

Supplementary Table 2. Cohort characteristics stratified by diagnostic label. RR is the clinical respiratory rate.
* denotes a potentially predictive difference between diagnostic groups. Models may detect age-related breathing rate differences rather than pathological signatures. This is confirmed by the good performances of the baseline models, which only use age and RR as features. Otherwise, pathological classes are similar in terms of mean SpO2 (a marker of severity) and coughing prevalence.

	Control	Pneumonia	Wheezing disorder	Bronchiolitis
Age, yrs (mean)	3.1	2.6	4.3	**0.3 ***
—CI95%	*2.6—3.7*	*2.2—3.0*	*3.7—4.9*	*0.3—0.4*
RR, resp/min (mean)	30.5	**42.9***	**38.1***	**55.0***
—CI95%	*29.1—31.8*	*40.4—45.4*	*35.9—40.3*	*52.9—57.1*
Temp, °C (mean)	36.7	38.2	37.3	37.3
—CI95%	*36.6—36.8*	*38.0—38.5*	*37.1—37.5*	*37.1—37.4*
SpO2 (mean)	98.6	95.5	95.0	95.5
—CI95%	*98.4—98.8*	*94.9.0—96.1*	*94.4—95.6*	*94.7—96.2*
Cough (%)	0.6	94.7	96.2	89.2

Supplementary Table 3. Hyper-parameters of Baseline models. These hyper-parameters led to the best average AUROCs on the validation sets of the nested cross-validation following a grid search. All models use L2 regularization. C is the inverse of the regularization parameter as defined in Sklearn's version 0.24.1 [2]. *Solver* is the optimization method. The optimization methods' names are the ones used in Sklearn.

Binary Model	C	*Solver*
Control	0.1	newton-cg
Pneumonia	0.1	sag
Wheezing disorder	10.0	liblinear
Bronchiolitis	1.0	newton-cg

Supplementary Table 4. Sensitivity breakdown of binary models. Each binary model *(first column)* classifies their **Target** (highlighted in bold) vs the other classes. Here, we stratify the rest of the classes to show the sensitivity of that model for correctly classifying the other classes as 'other'. Results are for the external test set.

	**Sensitivity per target**			
Binary Model	Control	Pneumonia	Wheezing disorder	Bronchiolitis
Control	**0.770**	0.776	0.818	1.000
Pneumonia	0.852	**0.469**	0.848	0.792
Wheezing disorder	0.639	0.653	**0.636**	0.958
Bronchiolitis	0.984	0.857	0.909	**0.500**

Supplementary references

1. Rocha, B. *et al.* *A respiratory sound database for the development of automated classification*, 33–37 (Springer, 2017).
2. Pedregosa, F. *et al.* Scikit-learn: Machine learning in Python. *Journal of Machine Learning Research* **12**, 2825–2830 (2011) .
