# Supplementary material for: DeepBreath—automated detection of respiratory pathology from lung auscultation in 572 pediatric outpatients across 5 countries
Source: NPJ Digit Med. 2023 Jun 2;6:104. doi: 10.1038/s41746-023-00838-3 (PMC10238513; doi:10.1038/s41746-023-00838-3)
Supplement: Supplementary file 2 — Reporting Summary [file 41746_2023_838_MOESM2_ESM.pdf]

nature portfolio

Corresponding author(s): Mary-Anne Hartley

Last updated by author(s): 14.10.2022

Reporting Summary

Nature Portfolio wishes to improve the reproducibility of the work that we publish. This form provides structure for consistency and transparency in reporting. For further information on Nature Portfolio policies, see our Editorial Policies and the Editorial Policy Checklist.

Statistics

For all statistical analyses, confirm that the following items are present in the figure legend, table legend, main text, or Methods section.

n/a	Confirmed	
☐	☒	The exact sample size (*n*) for each experimental group/condition, given as a discrete number and unit of measurement
☐	☒	A statement on whether measurements were taken from distinct samples or whether the same sample was measured repeatedly
☐	☒	The statistical test(s) used AND whether they are one- or two-sided<br>*Only common tests should be described solely by name; describe more complex techniques in the Methods section.*
☐	☒	A description of all covariates tested
☐	☒	A description of any assumptions or corrections, such as tests of normality and adjustment for multiple comparisons
☐	☒	A full description of the statistical parameters including central tendency (e.g. means) or other basic estimates (e.g. regression coefficient) AND variation (e.g. standard deviation) or associated estimates of uncertainty (e.g. confidence intervals)
☒	☐	For null hypothesis testing, the test statistic (e.g. *F*, *t*, *r*) with confidence intervals, effect sizes, degrees of freedom and *P* value noted<br>*Give P values as exact values whenever suitable.*
☒	☐	For Bayesian analysis, information on the choice of priors and Markov chain Monte Carlo settings
☒	☐	For hierarchical and complex designs, identification of the appropriate level for tests and full reporting of outcomes
☒	☐	Estimates of effect sizes (e.g. Cohen's *d*, Pearson's *r*), indicating how they were calculated

Our web collection on statistics for biologists contains articles on many of the points above.

Software and code

Policy information about availability of computer code

Data collection	Digital lung auscultations were recorded in WAVE (.wav) format with a Littmann 3200 electronic stethoscope (3M Health Care, St. Paul, USA) using the Littmann StethAssist proprietary software v.1.3 and Bell Filter option. The stethoscope has a sampling rate of 4,000 Hz and a bit depth of 16 bits.
Data analysis	The code used to train and evaluate DeepBreath is available on GitHub at https://github.com/epfl-iglobalhealth/DeepBreath-NatMed22. We developed custom Python code (version 3.8) using the following libraries:<br>torch (version 1.10.0, https://pytorch.org), torchaudio (version 0.10.0, https://pytorch.org/audio/stable/index.html), torchlibrosa (version 0.0.9, https://github.com/qiuqiangkong/torchlibrosa), scipy (version 1.7.2, https://scipy.org), pydub (version 0.25.1, https://pypi.org/project/pydub), pandas (version 1.3.4, https://pandas.pydata.org), matplotlib (version 3.4.3, https://matplotlib.org), audiomentations (version 0.19.0, https://pypi.org/project/audiomentations), for processing the WAVE files, performing statistical analyses and producing figures.<br>This is directly available in the code repository.

For manuscripts utilizing custom algorithms or software that are central to the research but not yet described in published literature, software must be made available to editors and reviewers. We strongly encourage code deposition in a community repository (e.g. GitHub). See the Nature Portfolio guidelines for submitting code & software for further information.

Data

Policy information about availability of data

All manuscripts must include a data availability statement. This statement should provide the following information, where applicable:

- Accession codes, unique identifiers, or web links for publicly available datasets
- A description of any restrictions on data availability
- For clinical datasets or third party data, please ensure that the statement adheres to our policy

Anonymized data are available upon reasonable request (alain.gervaix@huge.ch) which matches the intention to improve the diagnosis of paediatric respiratory disease in resource-limited settings. The audio used in the study are not publicly available to protect participant privacy. Unlimited further use is not permissible from the informed consent. The full code and test sets are available at the following GitHub repository: https://github.com/epfl-iglobalhealth/DeepBreath-NatMed22. Additionally, the model is available on DISCO https://epfml.github.io/disco/#/ to provide access to federated and decentralised collaborative training.

Human research participants

Policy information about studies involving human research participants and Sex and Gender in Research.

Reporting on sex and gender	The terms "sex" and "gender" have been used appropriately in this manuscript. Gender was not captured in the current study as all patients were paediatric and many under the age of coherent expression and the emergence of secondary sexual characteristics (under 60months).<br>The cohorts are sex balanced to ensure representation.
Population characteristics	A detailed breakdown of participants stratified by geographic site and diagnostic label are provided in Table 1.<br>Distributions of age and respiratory rates between cases and controls are provided in Figure S1.<br>The train-test splits were balanced by geographic site and diagnostic label.
Recruitment	Participants were recruited sequentially according to the afore mentioned criteria. The cohort may exclude some more severe cases who would be prioritized for care and referral.
Ethics oversight	The study is approved by the Research Ethics Committee of Geneva and local research ethics boards in each participating country. All patient's caregivers provided written informed consent.

Note that full information on the approval of the study protocol must also be provided in the manuscript.

Field-specific reporting

Please select the one below that is the best fit for your research. If you are not sure, read the appropriate sections before making your selection.

☒ Life sciences ☐ Behavioural & social sciences ☐ Ecological, evolutionary & environmental sciences

For a reference copy of the document with all sections, see nature.com/documents/nr-reporting-summary-flat.pdf

Life sciences study design

All studies must disclose on these points even when the disclosure is negative.

Sample size	A detailed breakdown of participants stratified by geographic site and diagnostic label are provided in Table 1.<br>A total of 572 patients were recruited in the context of a multi-site observational cohort study using a standardised acquisition protocol. Selection criteria aimed to recruit patients below the age of 16 presenting at paediatric outpatient facilities who had suspected lower respiratory tract infection, acute asthma or obstructive bronchitis. Patients with known chronic underlying respiratory disease (e.g. fibrosis) or heart failure were excluded.<br>In total, 71\% (n=407/572) were clinically diagnosed cases with one of three diagnostic labels: (i) pneumonia, (ii) wheezing disorders, or (iii) bronchiolitis. The remaining 29\% (n=165/572) were age- and sex-balanced controls with no respiratory symptoms, consulting at the same emergency unit for other complaints.<br>Distributions of age and respiratory rates between cases and controls are provided in Figure S1.
Data exclusions	Not applicable. All available patients from the listed collection sites who had a diagnostic label and complete digital auscultations were used.
Replication	Several independent data scientists have reproduced these results and validated the code. We have made the code available, with clear documentation. We also aim to make the model available for immediate simple deployment and further training using a distributed learning platform: DISCO: https://epfml.github.io/disco/#/
Randomization	Not applicable (this is an observational cohort study with sequential recruitment). However the train-test splits for model development were performed randomly (ensuring balanced representation of disease labels and geography)

Blinding	Not applicable (this is an observational cohort study with sequential recruitment). However the model, was never exposed to the internal or external test set during training.

Reporting for specific materials, systems and methods

We require information from authors about some types of materials, experimental systems and methods used in many studies. Here, indicate whether each material, system or method listed is relevant to your study. If you are not sure if a list item applies to your research, read the appropriate section before selecting a response.

Materials & experimental systems

n/a	Involved in the study
☒	☐ Antibodies
☒	☐ Eukaryotic cell lines
☒	☐ Palaeontology and archaeology
☒	☐ Animals and other organisms
☐	☒ Clinical data
☒	☐ Dual use research of concern

Methods

n/a	Involved in the study
☒	☐ ChIP-seq
☒	☐ Flow cytometry
☒	☐ MRI-based neuroimaging

Clinical data

Policy information about clinical studies
All manuscripts should comply with the ICMJE guidelines for publication of clinical research and a completed CONSORT checklist must be included with all submissions.

Clinical trial registration	n/a not interventional
Study protocol	https://github.com/epfl-iglobalhealth/DeepBreath-NatMed22
Data collection	All participants were recruited between 2016 and 2020 at the outpatient departments of six hospitals across five countries (120 in Geneva, Switzerland; 283 in Porto Alegre, Brazil; 31 in Dakar, Senegal; 79 in Yaoundé, Cameroon; and finally 59 from Rabat and Marrakesh in Morocco).<br>Detailed summary statistics are provided in Table S1
Outcomes	All diagnoses are validated by two medical doctors. Pneumonia is diagnosed radiologically where possible or by the presence of audible crackles and/or febrile respiratory distress. 90% of the pneumonia cases were reported as bacterial, and the rest as viral. These are grouped into a single category of pneumonia due to the notorious difficulty of distinguishing between them objectively. Bronchiolitis and wheezing disorders (comprising asthma and obstructive bronchitis) are diagnosed clinically.
